# The emergence of use of a rake-like tool: a longitudinal study in human infants

**DOI:** 10.3389/fpsyg.2014.00491

**Published:** 2014-05-23

**Authors:** Jacqueline Fagard, Lauriane Rat-Fischer, J. Kevin O'Regan

**Affiliations:** Laboratory Psychology of Perception, CNRS UMR 8242 – Université Paris DescartesParis, France

**Keywords:** tool use, infants, spatial contiguity, longitudinal, observational learning

## Abstract

We describe the results of a longitudinal study on five infants from age 12 to 20 months, presented with an out of reach toy and a rake-like tool within reach. Five conditions of spatial relationship between toy and rake were tested. Outcomes and types of behavior were analyzed. There were successes observed around 12 months in the condition of spatial contiguity between rake and toy, but these could not be interpreted as corresponding to full understanding of the use of the rake. At this age and for the following months, in the conditions involving spatial separation between rake and toy, infants' strategies fluctuated between paying attention to the toy only, exploring the rake for its own sake, and connecting rake and toy but with no apparent attempt to bring the toy closer. Only between 16 and 20 months did infants fairly suddenly start to intentionally try to bring the toy closer with the tool: at this stage the infants also became able to learn from their failures and to correct their actions, as well as to benefit from demonstration from an adult. We examine the individual differences in the pattern of change in behaviors leading to tool use in the five infants, and find no increase in any one type of behavior that systematically precedes success. We conclude that sudden success at 18 months probably corresponds to the coming together of a variety of capacities.

## Introduction

Tool use is the ability to use one object (the “tool”) to manipulate other objects, and hence move beyond the limits imposed by the length of one's limbs or the type of one's end-effector (Nabeshima et al., 2006). Tool use has often been recognized as an important step during evolution (van Schaik et al., 1999), and as a marker of the evolution of human intelligence (Wynn, 1985). Its importance as a milestone in human development has also long been recognized (Piaget, 1952) and is still emphasized (“a royal road to the study of problem solving,” Keen, 2011, p. 2). And more recently, understanding the basis of tool use has come to be seen as fundamental for robotics (Nabeshima et al., 2006).

Curiously, the development of tool use in human infants has received relatively little interest until recently (see Keen, 2011, and Greif and Needham, 2011, for reviews). In addition, most of the existing studies have been concerned more with describing stages of skill development or factors that induce success, than with suggesting precise learning mechanisms. Furthermore, very few of these studies have been longitudinal.

One possible exception, both for being longitudinal and for looking for mechanisms, is Piaget. Piaget first described “la conduite du bâton” (stick behavior) in 1952. He noticed that his children started to use a stick to move far away objects by the end of the first year. Piaget had noted that his son Laurent discovered the use of the stick “almost without trial and error” (Piaget, 1952, p. 290). The question asked by Piaget in 1952 was “how to explain the transition from trial and error to invention, from motor scheme to representative scheme.” Another longitudinal study is that of Connolly and Dalgleish (1989). In this study, Connolly and Dalgleish observed two groups of infants, aged 11 and 17 months, at monthly intervals over a 6-month period, as they tried to use a spoon. However, Connolly and Dalgleish were more interested in changes in the shape of the movement leading to expertise (hand use, grip pattern, spoon trajectory) than in the underlying mechanisms leading to an understanding of the use of the spoon to retrieve the food.

Another exception, not for being longitudinal but for being interested in underlying mechanisms, is a study by Bruner, who observed how children progress from one level of organization to the next when using a primitive form of tool, a lever with fixed fulcrum (Koslowski and Bruner, 1972). From their cross-sectional study of 12-to-14, 14-to-16, and 16-to-23-month-old children learning to use the lever to obtain a toy attached to the end of the lever, Koslowski and Bruner extracted some principles to explain how the child progresses from one level of organization to the next. For them, the transition seems to involve the child concentrating on the two individual components of the task (how the rotation of the lever affects the position of the goal, and how the child can effect a rotation of the lever). Once each of the components has been modularized and is less attention-demanding, the child becomes able to attend simultaneously to the movement of both the lever's goal end and its hand end. This allows them to finally envision the solution to the problem.

However, Koslowski and Bruner's lever task is not a real tool use task *stricto sensu*, because the tool is not completely independent of the object to be retrieved. In Koslowski and Bruner's task, the child had to select among several means the appropriate one for achievement of the end state. This kind of task, involving the planful execution of a sequence of steps to achieve a goal, is referred to as a means-end task, and has been amply studied since Piaget (1952), mostly with cross-sectional studies. Examples of means-end tasks are for instance pulling a support to retrieve an object resting on it (Willatts, 1999), pulling a string to retrieve an object attached to it (Uzgiris and Hunt, 1975; Brown, 1990), pulling one of two strings to retrieve an object attached to it one of the strings (e.g., Chen et al., 1997). Tool use, where the tool is completely independent of the toy to be retrieved, as in the study presented here, may be considered as belonging to an extended category of means-end tasks. However it differs from the simplest means-end tasks by the complete spatial discontinuity between the tool and the object to be retrieved.

There have been some cross-sectional studies on tool use, with the tool being independent of the object to be acted upon. For instance, use of a spoon, already the object of Connoly and Dalgeish's longitudinal study, was also considered in a cross-sectional study focusing on progress in action planning (McCarty et al., 1999). Nine-, 14-, and 19-month-old children were given a spoon presented in such a way that the bowl part was on the side of the preferred hand. Only the 19-month-olds anticipated the problem and directly grasped the handle with the ipsilateral non-preferred hand, whereas younger infants used their ipsilateral (preferred) hand to grasp the bowl part of the spoon or the handle part with an awkward movement. Another spoon study, also cross sectional, focused on the role of prior experience on tool use (Barrett et al., 2007). In this study, the task was to turn on a light inside a box. The infants showed much less flexibility in grasping the spoon in an unusual way (different from the habitual grasp of the spoon) demonstrated by the experimenter than in grasping a new tool. For the authors, this meant that “rather than learning about tool function… infants learn about which part of the tool is meant to be held… ” (op. cit., p. 352).

Infants generally have ample opportunity to familiarize themselves with use of a spoon, so it is not the best tool on which to study the emergence of tool use from scratch. A few cross-sectional studies have investigated what factors contribute to the difficulty to use a new tool to get an out-of-reach object. They all stress that difficulty in tool use increases with an increasing spatial gap between the tool and the object to be acted upon (Bates et al., 1980; van Leeuwen et al., 1994), or more generally with an increasing number of steps needed to achieve the required result (Smitsman and Cox, 2008). In their 1980 study, Bates et al. compared 40 10-month-old infants retrieving an out-of-reach toy placed either on a cloth, at the end of a string, or at different positions near three kinds of tool likely to help the children retrieve it (hoop, crook or stick). The conditions where toy and tool are physically linked (“unbreakable contact”) were succeeded most, followed by the conditions in which there was breakable contact (toy placed against/inside the hoop or the curved part of the crook). The conditions with no contact (toy beside the crook or the stick) were succeeded least. The authors concluded that at 10 months solving the problem is easier when the link between the tool and the toy is suggested by the spatial array. Van Leeuwen et al. proposed that the role of spatial contact between tool and toy in helping infants solve the problem was partly linked to the number of mental transformations that the infants must perform to imagine the solution (“number of elements to be integrated,” 1994, p. 189).

In summary from this brief review of the literature, we can conclude that spatial proximity, number of transformations, and also familiarity with the tool are important factors, and that planning of action improves with practice. However, we still lack an understanding of the cognitive mechanisms that underlie the acquisition of tool use ability in the course of the second year. In particular, we cannot as yet answer Piaget's question as to whether tool use appears through sudden insight or emerges gradually through progressive familiarization with tool affordances. According to Lockman (2000), tool use emerges from a long period of object manipulation that familiarizes infants with the use of an object to interact with other objects. On this view, the progressive discovery of the various affordances of an object allows infants to later ascertain which affordance will solve their problem. This ecological view contrasts with the more radical view that tool use results from sudden insight (Köhler, 1927).

To more precisely explore the mechanisms underlying the acquisition of tool use, and in particular to ascertain whether this acquisition occurs gradually or through sudden insight, a longitudinal study is called for. We decided to take a small number of infants and study their evolution from ages 12 to 20 months on a regular basis, carefully analyzing their behavior longitudinally as they learned to use a rake-like tool to obtain an out-of-reach toy. The questions we posed were: why is spatial proximity an important factor? What will allow infants to understand the affordance of the rake in conditions of no spatial contact: Observation of their success in easier conditions of spatial contact? Exploration of the rake? Trial and error? Observation of a demonstration? Sudden insight?

Regularly following a small number of infants during the months preceding the acquisition of a skill has in the past proven to be a good way to gather useful information on mechanisms underlying skill acquisition (Piaget, 1952; Thelen et al., 1993). It is also one way to look at individual trajectories as well as common patterns.

## Methods

We constructed a T-shaped rake-like tool made out of white cardboard with a 20-cm-long handle. We used a selection of small toys that we had previously determined to be interesting to children in a day-care nursery. Infants were comfortably seated at a testing table during the whole session. They were either on the parent's lap or, at older ages, in a high chair. By using a white rake, we ensured that the rake was not itself strongly attractive, as compared to the visually highly salient toys that we used so as to attract the attention and trigger the desire of the infants.

**Figure 1:**
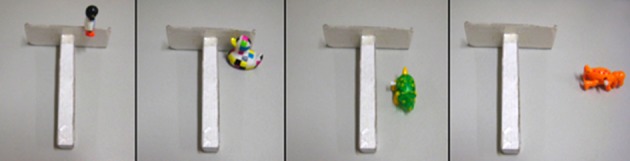
**Rake and toys in the different conditions of testing from C1 to C4**. (C5 is not shown: the toy is placed too far to be reached and the rake is handed directly to the infant).

Five infants (two girls and three boys) were observed regularly in five conditions: toy on top of rake, attached to it (C1), toy inside/against the rake (C2), toy inside the rake but not against it (C3), toy to the side of the rake (C4) (see Figure 1), and rake handed to the infant (C5). The toy was always just out of arm's reach.

All infants were brought in at 11 months for familiarization with the experimental room and the experimenters. Testing started when the infants were 12 months old. They were tested every month until they could use the rake with success (16 months for one infant, 18 months for three of the infants, 20 months for the fifth). Mean age was 12 months at session 1 (Ses1), 13 months 1 day at Ses2, 14 months 4 days at Ses3, 15 months 6 days at Ses4, 17 months 1 day at Ses5, and 18 months 8 days at Ses6. Four infants took part in 6 sessions. Infant 2 (I2) was seen at 11 months but missed sessions 1 and 2 for family reasons; we kept him in the study for two reasons: first because of the small number of infants; second, because we thought it interesting to compare his performance on his first session with that of the other infants of the same age but who had had two practice sessions. Infant 5 succeeded at session 4 (16 months) and was seen again at 20 months to check the stability of performance. Condition C1 was only tested once at the beginning of each session since it does not represent a challenge for infants at the ages tested here and it is not strictly speaking tool use. Results from this condition are briefly mentioned at the beginning of the Results section but not included in later analyses. The other conditions were tested several times per session. Since this study was exploratory, we decided to test the infants for as long as they were willing to participate, rather than to have exactly the same number of trials per infant. We checked that the difference in number of trials between infants was not related to a difference in age of success. At the beginning of each session, the order of presentation was from C1 to C5, but when an infant was willing to continue, conditions C2, C4, and C5 were retested in unsystematic order. After the first failure on C4 and C5, a demonstration was provided by one of the adults present in the room, either a parent or an experimenter. A demonstration consisted in two or three repetitions of showing the infant, while he or she was looking at the toy/rake, how to bring the object toward himself or herself. The demonstrator always showed how to use the rake from the infant's perspective and accompanied the demonstration by encouraging small talk (“*Look how you can do it, look what I do to get the toy…*”). The rake was then either put back in front of the infant (C4) or handed directly to the infant (C5). There were 389 trials in all, and between 1 and 3 demonstrations per session. A trial was terminated if the infant did not try to obtain the toy within one minute, or after failing to retrieve the toy. After getting the toy, the infants were allowed to play with it for about one minute.

The research was approved by a local ethical committee.

### Coding of behaviors

We first coded elementary behaviors in each condition of each trial for each of the five infants for the 362 trials of conditions C2–C5. These elementary behaviors involved looking (infant looks at toy, at rake, at adult, or elsewhere); pointing toward toy (with bare hand or with rake); grasping the rake (after touching it by chance, spontaneously, encouraged by the experimenter, or put in the baby's hand by the experimenter), moving the rake (rakes it or lifts it from the table with inside or outside lateral movements, or with a straight movement toward himself; makes a detour around the object or not); refusing the rake (refuses it when handed by experimenter, places it on table, throws it away); manipulating the rake *per se* (puts rake into mouth, bimanually explores rake) or on the table (swipes table, rubs table, hits table); manipulating the rake in connection with the toy with no clear intention to bring it back (hits toy or pushes toy with rake); interacting with the adult, clearly asking for help (gives rake to adult, takes the adult's hand and places it on rake); and manipulating the rake with clear intention to bring the object back (brings object to hand with rake; with wrong movements, peculiar but effective movements, or direct movements; prepares the second hand while raking the toy with the first hand). These elements of behavior occurred together in several ways during trials, leading to a count of 26 whole-trial behaviors among all 362 trials (see Table 1). The whole-trial behaviors are grouped into categories as a function of the level of performance they reflect and these categories give a raw score. The notation NT (No Try) means that the child did not try either to retrieve the toy or to explore the rake. T (Toy) indicates that the child was interested in the toy; R (Rake) means that the child was interested in the rake but in neither case was s/he interested in their interaction. T+R indicates that the child was interested in the interaction between rake and toy without showing a clear intention to retrieve the toy. S1 (Success level 1) indicates that the child appeared to show clear understanding of the rake as a possible tool to retrieve the toy but did not yet know how to use the rake. S2 (Success, top level) means that the child clearly knew how to retrieve the toy with the rake.

**Table 1:** **Different strategies observed during a whole trial (in a few trials two strategies, or more rarely three, occurred in succession)**.

**Whole-trial behaviors**
**NO TRY (NT)**
1. Grasps rake, gets rid of it, stops being interested (*rake is grasped here without being the focus of attention)*
2. Looks at toy, looks at rake, looks at adult, doesn't do anything
3. Refusal
**T: BEGGING FOR TOY AND NOT USING RAKE AFTER ITS GRASPING LEADS TO FAILURE**
4. Points to toy and refuses or ignores the rake
5. Points to toy, then grasps rake (either spontaneously or encouraged by the experimenter), points again toward toy with other hand
6. Grasps rake, the toy does not come, does not try again with the rake, may then point to toy with bare hand
7. Grasps rake, gets rid of it (throws it away, places it on the table), and points to the toy
8. Looks at toy, pulls rake while looking at toy, stops action with rake when sees that toy does not come, points to toy
**R: EXPLORING RAKE BUT NOT USING IT IN CONNECTION WITH THE TOY**
9. Points to toy, then grasps rake and plays with it (puts into mouth or rubs, swipes, hits, etc. on table)
10. Grasps rake, interested in rake only (puts into mouth or rubs, swipes, hits, etc. on table)
11. Grasps rake, swipes table with it and sweeps toy away by accident
12. Grasps rake, plays with it and then rejects it, may be interested in toy again
**T+R: USING RAKE IN CONNECTION WITH TOY BUT NOT FOR RETRIEVAL**
13. Points to toy, then grasps rake (spontaneously or encouraged by the experimenter) and touches or pushes toy with it
14. Grasps rake, touches or pushes object with rake
15. Grasps rake (after pointing first to toy or not), points to toy with rake
**S1: USING RAKE FOR RETRIEVAL: TRIAL AND ERROR, DIFFICULT OR PARTIAL SUCCESS, OR ONLY AFTER DEMONSTRATION**
16. Grasps rake, moves rake, tries to bring back toy, partial success
17. Grasps rake (after pointing first to toy or not), retrieves or tries to retrieve toy after demonstration
18. Grasps rake after being encouraged (after pointing first to toy or not), moves rake and retrieves toy with it
19. Grasps rake (after pointing first to toy or not), awkward movements to bring toy to hand, success
20. Grasps rake (after pointing first to toy or not), retrieves toy after several attempts
**S2: USING RAKE FOR RETRIEVAL: INTENTIONAL MATURE SUCCESS**
21. Grasps rake, moves rake to retrieve toy, success
**AMBIGUOUS CASES (NOT INTERPRETABLE, THUS NO SCORE)**
22. Points to toy, hand on rake more or less by chance, grasps rake, rakes with it, toy comes by contingency (at C2 or C3)
23. Points to toy then grasps rake encouraged by experimenter and brings the toy to hand possibly by contingency (at C2 or C3)
24. Points to toy, grasps rake spontaneously, retrieves toy possibly by contingency (at C2 or C3)
25. Grasps rake spontaneously, retrieves toy possibly by contingency (at C2 or C3)
26. Grasps rake (spontaneously or encouraged by the experimenter) and gives rake to adult or grabs adult's hand

*S1 and S2 were coded for C4 and C5 only, when the rake had first to be displaced laterally to be used*.

Notice that in our classification, the last category of behaviors, which we call “A” for “Ambiguous” (behaviors 22–26), has a special status. Behaviors 22–25 occurred in conditions C2 and C3 where it was possible for the child to succeed without any understanding of the functionality of the rake, as we shall see below. This is because, due to the physical position of the toy inside the rake, simply pulling the rake automatically brought the toy into reach. Success in this condition could thus be due to the contingency between rake and toy, and would not necessarily indicate understanding of the function of the rake: hence our coding of “Ambiguous.” Finally, there was also another behavior (26) that we could not interpret and that we have included in the “ambiguous” category: sometimes the infant simply grasped the rake and gave it to the adult. She may have done so because she wanted the adult's help and had understood that the rake was the key element, or because she wanted to get rid of the rake. This behavior was observed only eight times in all, in three of the infants.

### Data analyses

Each infant received a score for each trial, depending on the category it fit in: 0 (No Try), 1 (interested only either in the toy, or in the rake, T or R), 2 (using the rake in connection with the toy, not for retrieval, T+R), 3 (using the rake for retrieval but with difficult or partial success or only after demonstration, S1), or 4 (intentional spontaneous mature success, S2).

For some statistical tests we pooled C2 and C3, the two conditions without spatial gap, and C4 and C5, the two conditions with spatial gap.

For each significant effect of ANOVA, the effect size was calculated as partial eta^2^, using the formula: η^2^ = SS_effect_/SS_total_, where *SS*_effect_ = the sums of squares for sessions or conditions, and *SS*_total_ = the total sums of squares for sessions or conditions and errors.

## Results

### Retrieval of the toy as a function of condition and session

Before considering the detailed behaviors as classified in our detailed coding scheme, we present in this first section an analysis of overall success, including the ambiguous successes, at retrieving the toy. The results for overall success bear on 389 trials: 27 for C1, 89 for C2, 60 for C3, 118 for C4, and 95 for C5, in all. Most of the time the infants were interested in the task. They sometimes expressed frustration at not being able to get the toy, but they rarely refused a trial. NT (No Try) was coded for 31 trials (7.9%). NT never occurred in C1. For the four other conditions the percentage of NT did not change with condition (*p* = 0.52).

#### Toy attached to the rake (C1)

**Figure 2:**
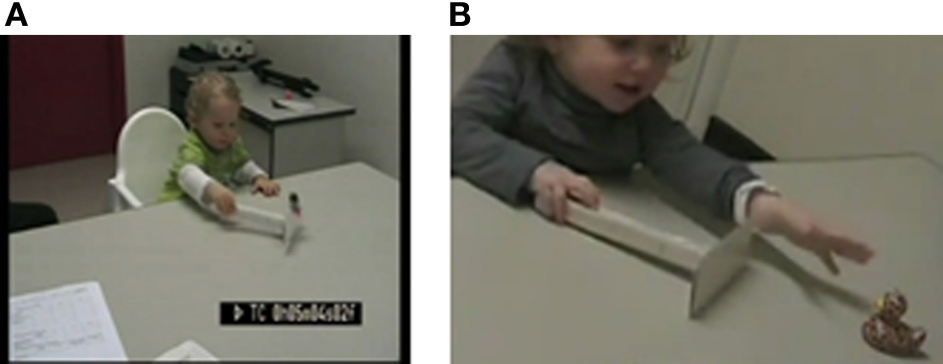
**(A)** Grasping the rake directly (C1), **(B)** Reaching/pointing toward the toy (C4).

When the toy was attached to the rake (C1), the infants grasped the rake without hesitation and then detached the toy from the rake (see Figure 2A). They almost never first reached or pointed toward the toy in this condition (see below results on pointing as first behavior and Figure 2B). All infants looked clearly at the toy from the start of their pulling movement. This shows that visual information sufficed for them to understand that the toy was connected to the rake. Success was always 100%, starting on the first trial.

#### Toy inside the rake (C2 and C3)

**Figure 3:**
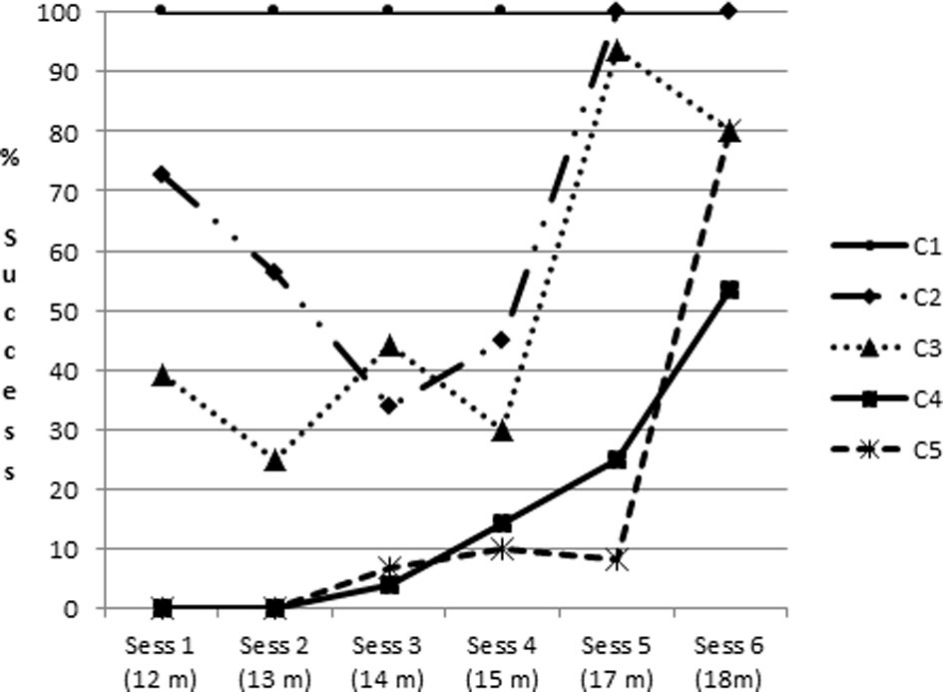
**Percentage of success as a function of condition and session (ambiguous successes are included)**.

The rate of toy retrieval was high as of the first session, particularly for C2, as can be seen in Figure 3 which represents the mean percentage of success in which the toy was retrieved successfully. Rates of success for C2 and C3 did not differ significantly. In C2, in the first session infants most often immediately grasped the rake to make a raking movement leading to successful retrieval. When the toy was not against the rake (C3), these successful retrievals represented only 39% of trials.

The rate of toy retrieval in C2 showed a U-shaped form. After the first session and the rather stereotyped behavior seen in it (the majority of observed strategies were A25), infants demonstrated various behaviors in C2, as we shall see below. An ANOVA on the frequency of object retrieval in C2 as a function of session showed a significant and moderate effect [*F*_(5, 15)_ = 4.3, *p* < 0.02; partial η^2^ = 0.59]. An LSD *post-hoc* test indicated that the percentage of retrieval was almost significantly higher at the first compared to the third session (*p* = 0.06). The percentage of retrieval was significantly lower at sessions 2, 3, and 4 than at sessions 5 and 6. Percentage of success in C3 showed an increase across sessions but no statistics were calculated on C3 alone because of missing data.

#### Toy to the side of the rake (C4 and C5)

All infants younger than about 16 months failed to retrieve the toy when it was not inside the rake, except two infants who succeeded once in the third session but did not repeat it. Successes in C4 and C5 showed a rather sudden increase between sessions 5 and 6 (see Figure 3). In session 6, all five children succeeded in C4 and C5, although they still did not succeed on all trials, as can be seen in Figure 3. An ANOVA on the frequency of object retrieval in C4 and C5 combined (percentage of “S1” + “S2”) as a function of session showed a significant and large effect [*F*_(5, 15)_ = 15.9, *p* < 0.001; partial η^2^ = 0.73]. A LSD *post-hoc* test indicated that the percentage of retrieval was significantly different in session 6 as compared with all the other sessions, which did not differ significantly from each other. Interestingly, infant 2 who missed sessions 1 and 2, and is compared with the others for age (that is, he is included in session 3 at age 14 months as if it was his third session even though it was his first session) is well within the mean of all infants (see Figure 7 for individual results).

#### Comparison between conditions

In term of success, an ANOVA on the frequency of retrieval as a function of condition and session was calculated. For this calculation we used the mean frequency of success at C2 and C3, the mean frequency of success at C4 and C5 and compared both of them to success at C1 (See Table 2). Results show a significant main significant and large effect of condition [*F*_(2, 30)_ = 94.2, *p* < 0.0001; partial η^2^ = 0.84], a significant small main effect of session [*F*_(5, 30)_ = 11.2, *p* < 0.001; partial η^2^ = 0.80], and a significant large effect of condition x session interaction [*F*_(10, 30)_ = 6.5, *p* < 0.0001; partial η^2^ = 0.84]. A LSD *post-hoc* test shows that the condition effect is due to a difference between all conditions, C1 being better than C2–C3, itself better than C4–C5. For the session effect, it is due to a difference between sessions 1, 2, 3, 4 on one side and 5 and 6 on the other side. The first four sessions do not differ significantly from each other. The difference between sessions 5 and 6 almost reach significance (*p* = 0.05). A LSD *post-hoc* analysis on the condition x session interaction indicates that condition 1 is better than conditions 2–3 at the first 4 sessions only, and better than conditions 4–5 at all sessions, and that conditions 2–3 are significantly better than conditions 4–5 at all sessions.

**Table 2:** **Mean success (%) as a function of session and condition (pooling C2–C3 and C4–C5)**.

	**Ses1**	**Ses2**	**Ses3**	**Ses4**	**Ses5**	**Ses6**
C1	100	100	100	100	100	100
C2–C3	50	33.4	38.7	38.8	95.8	90
C4–C5	0	0	7.5	13.7	13.2	74.8

**Figure 4:**
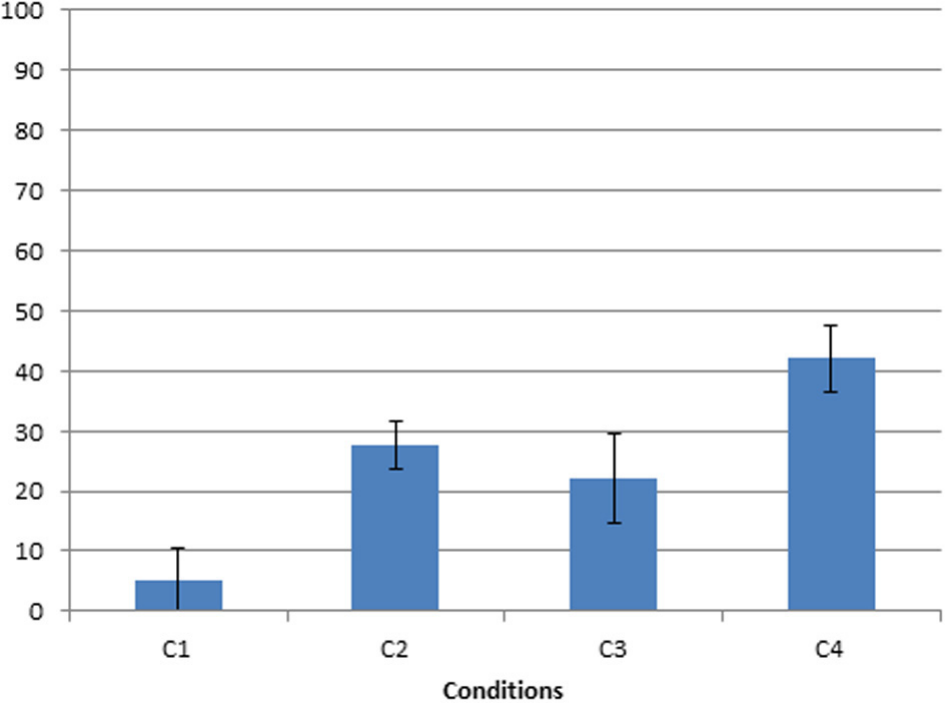
**Frequency of pointing first toward the object as a function of condition**.

Another analysis that we did on all conditions before moving to the more qualitative analyses of strategies concerns reaching toward / pointing to the toy as a first behavior. Infants frequently pointed to the toy before grasping the rake. As already mentioned, they almost never did it in condition C1 when the toy was attached on the rake. Pointing as a first behavior increased with task difficulty. An ANOVA on the percentage of reaching/pointing as a function of condition (C5 excluded, since in this condition the rake was handed directly to the infant) indicated a significant and large effect of condition on reaching/pointing [*F*_(3, 12)_ = 12.8, *p* < 0.001; partial η^2^ = 0.73]. An LSD *post-hoc* test indicated that the percentage of reaching/pointing was significantly lower in C1 than in all other conditions, and lower in C3 than in C4 (see Figure 4).

In conclusion, as of 12 months of age, retrieval of the toy was always successful when the toy was attached to the rake (C1), often successful when the toy was inside and against the rake when infants were 12 and 13 months old (C2) but less so on the next two sessions, and not successful at all when the toy was to the side of the rake until 16–20 months of age depending on the infants (C4–C5). Thus, early successes in C2 did not appear to help much in allowing the infants to understand how to use the tool, since these early successes were followed by many failures in C2 and by almost total failure in C4 and C5. In order to get cues to understand the U-curve shape observed in C2 and the relatively sudden onset of success observed in C4 and C5, and to answer our other questions (Why is spatial proximity an important factor? What helps infants understand the affordance of the rake in conditions of no spatial contact: Exploration of the rake? Trial and error? Observation of a demonstration? Sudden insight?), we shall undertake a finer analysis of behaviors as a function of condition and session. This is the purpose of the following section.

### Finer analysis of behaviors as a function of condition and session: what do these behaviors tell us about infants' understanding of the rake's functionality?

#### Toy inside the rake (C2)

In the following paragraphs we shall analyse more finely the behaviors observed in condition C2 in order to try to understand the origin of the U-shaped curve in retrieval rate observed over the successive sessions.

**Figure 5:**
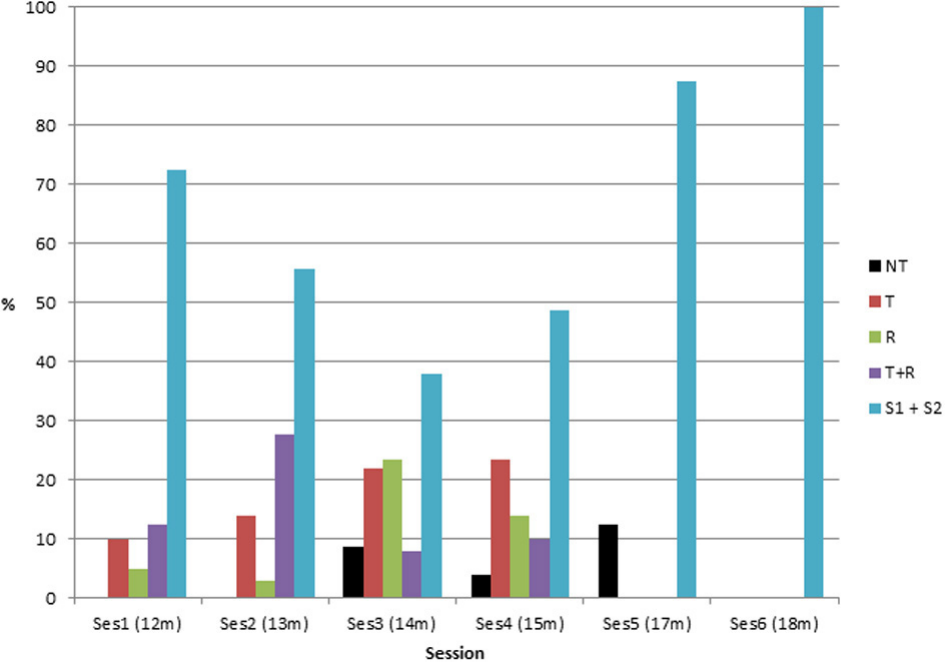
**Percentage of the different categories of behavior in C2 as a function of session**. (NT, refusal; T, interested in toy only; R, interested in rake only; T+R, interested in connection between rake and toy but not for retrieval; S1 + S2, partial or total success at toy retrieval).

As mentioned above, in Session 1 the most frequent behavior was elementary behavior A25 (60% as a mean for all infants), in which the child almost immediately grasps the rake and pulls it. Because the toy is spatially inside the tool, the toy generally comes along with the rake, and the child is able to retrieve it. This stereotyped direct pulling of the rake observed in session 1 for C2 decreases in frequency in sessions 2 (46.7%) and 3 (23.3%), being replaced by more varied behaviors in the next three sessions. By then, infants often pointed to the toy before pulling the rake (behaviors A23 and A24) or they started to rake the toy but the object was not brought near enough to be retrieved, they did not use a further raking movement to retrieve the toy and instead pointed to the toy with the empty hand (behaviors 5–7). Another frequent behavior was grasping the rake and playing with it (behaviors 13–15). More generally at this stage infants frequently took an interest only in the toy (reaching/pointing to the toy while ignoring the rake or after discarding it), or the rake (exploring the rake by itself, putting it into the mouth, rubbing, sweeping or hitting the table with it) (see Figure 5). Connecting rake and toy not for retrieval (touching or hitting it), was not often observed, except for one infant who used it from the first session.

We interpret all these behaviors typical of the few sessions following the first one as showing that the high rate of toy retrieval observed in the first session did not reflect a real understanding of the rake's functionality. There were three main reasons why we consider the first successes at C2 as ambiguous/uninformative and not reflecting a clear understanding of the affordance of the rake: the first is that the rate of toy retrieval decreased marginally significantly from session 1–3. The second reason is that when the infants started to rake the toy but failed to bring it close enough to grasp it, they never tried a second time to pull the toy with the rake: instead, they discarded the rake and pointed toward the toy with the empty hand. The third reason is that in several cases during the second to fourth session the infants did not pull the rake on the table but grasped it and lifted it over or around the toy before pointing toward the toy with the empty hand (see Video S1). In order to understand the origin of this pattern of behaviors, we may suppose the following. Infants may have grasped the rake as their first action either because it was the closest object or because they believed the toy to be attached to it as in C1 (and it may have taken them some time to realize that this was not the case). In any case, because the toy was touching the rake or almost touching the rake, the simple pulling of the rake was enough to bring the toy closer most of the time. When, in the following sessions, rather than pulling the rake directly, infants moved the rake around the toy and then pointed to the toy, begging for it with their bare hands after getting rid of the rake, they may have been showing their interest in the toy but also that they, at this stage, knew that the rake and the toy were not connected. Such behavior may also show that they did not know that the rake could be used to bring an unconnected object closer. Ultimately however, by sessions 5 and 6, the rate of successful retrieval in C2 went up again, probably corresponding to a true understanding of the rake's affordance.

It is worth noting that there were interindividual differences in the way new behaviors replaced the systematic pulling of the rake in the first sessions. Infant 1 was more interested in the toy than in the rake and behavior “T” replaced “A” at the following sessions. Infant 2 frequently explored the rake by itself on the third session (his first session). Infant 3 was very interested in exploring the rake from the beginning, either in connection with the toy or alone. For him, behavior “T+R” was frequent especially at sessions 2–4. Infant 4 was the infant whose ambiguous successes in C2 decreased the least after the first sessions. For her, behaviors “T” and “R” were frequent in sessions 3 and 4. Infant 5 showed the lowest rate of ambiguous success at the first session and either pointed to the toy (T) or was interested in exploring the rake from the beginning (R).

In conclusion, observation of behaviors in condition C2 across sessions indicates that after the early successes of the first sessions, infants' behavior changed in sessions 2–4. Instead of immediately pulling the rake, they either pointed to the toy, sometimes after discarding the rake, or they grasped the rake and explored it. Thus, in those sessions, infants tended to pay attention either to the toy or to the rake but they seldom connected the two objects and when they did, it was not to retrieve the toy. These switches between different strategies across sessions are comparable to the overlapping wave patterns described by Chen and Siegler (2000) in their microgenetic study of tool use at 18–35 months of age.

If, as we suggest, the early successes in toy retrieval in condition C2 were only due to the physical proximity between rake and toy (so that any movement of the rake would tend to bring the toy closer), rather than to a true understanding of the rake's functionality, this may explain why there was no rapid transfer from “successes” in C2 to successes in C4 and C5.

We will next analyze behaviors in conditions C4–C5 in order to elucidate how children understood how to use the rake when the toy was clearly separated from the tool.

#### Toy not near the rake (C4 and C5)

We have seen that despite all their experience of success (expected or not) when the toy was inside the rake, when it was clearly separate from the rake (in C4 and C5) it took the infants several sessions and many trials to understand how to use the rake to retrieve the toy. In particular, it took the infants 23–35 trials in all in C4 and C5 (median: 28 trials) across 4–6 sessions (mean: 5) to succeed, and they were aged 16–20 months (mean 17.8 months) when they reached this stage. If the infants did not learn much from their own “unexpected” success in C2, then how did they learn to use the rake in conditions C4 and C5? By exploring the rake? By trial and error? By watching a demonstration by an adult? In the following section we explore these alternatives by checking which behaviors preceded success in C4 and C5.

***Exploring the rake alone and in connection with the toy***. In this section we ask whether exploring the affordances of the rake over successive sessions allowed the child to accumulate enough knowledge to finally make the link between rake and tool, and thereby accomplish the task.

**Figure 6:**
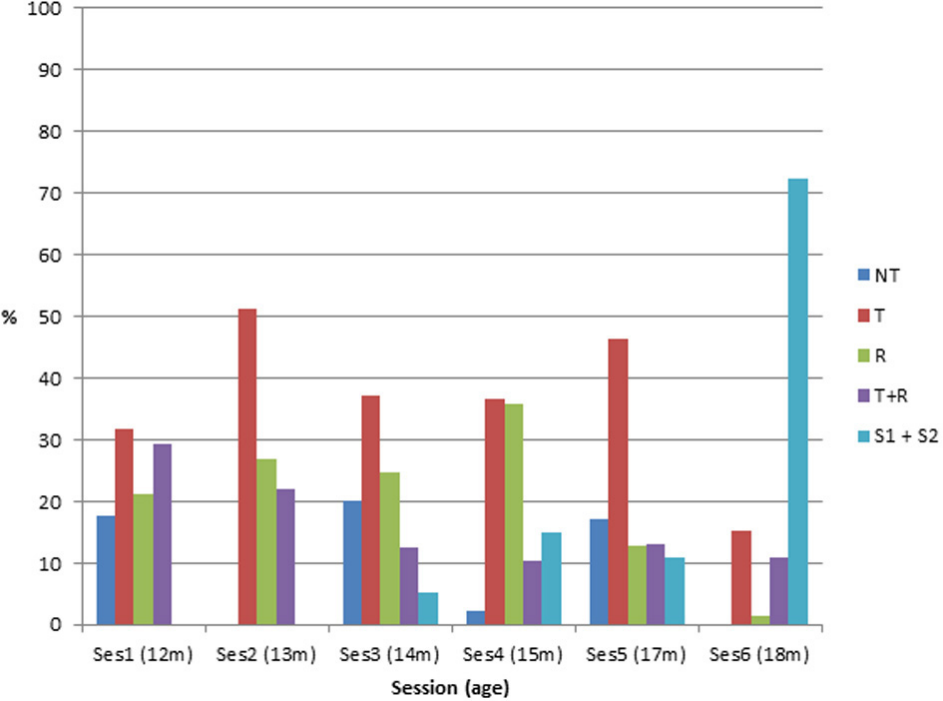
**Distribution of the five categories of behavior at C4–C5 as a function of session**. (NT, refusal; T, interested in toy only; R, interested in rake only; T+R, interested in connection between rake and toy but not for retrieval; S1 + S2, partial or total success at toy retrieval).

First of all, exploring the rake itself (“R”) was very frequent over the successive sessions (see Figure 6). It was the second most frequent behavior (20.5%, all sessions considered) after behavior “T” (36.4%). Connecting the rake with the toy (T+R) was less frequent (16.4%). Note that the “T+R” behaviors of the first sessions seemed not to be directed toward retrieving the toy (see Video S2), and were very different from behaviors 16 or 17 of S1 (see Table 1) observed in the last sessions where infants clearly connected the rake with the toy to try to retrieve it even though they failed. Hitting the toy with the rake seemed to be a game *per se* in the first sessions, and infants who used this strategy did not even grasp the toy systematically when it happened to come within reach after they hit it.

**Figure 7:**
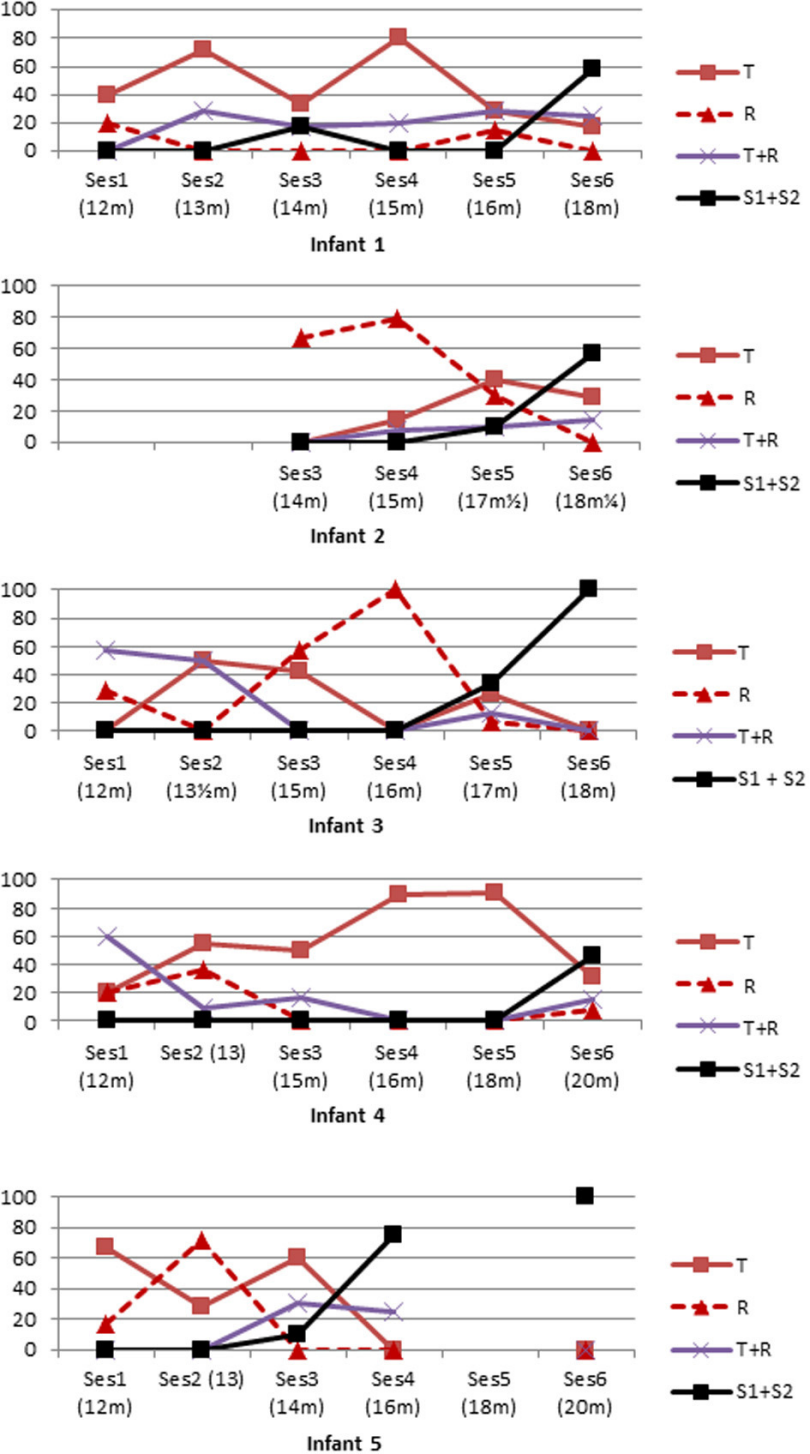
**Individual profiles in C4–C5**.

A second point is the following: individual patterns showed that all five infants fluctuated between the different strategies across sessions (see Figure 7). Sometimes they mostly pointed toward the toy, sometimes they mostly explored the rake, and at other times they mostly connected rake with toy. Doing statistics on the evolution of the different strategies across sessions would be misleading as it is clear that the five infants switched in different ways between pointing to the toy, exploring the rake, and connecting the rake with the toy (T+R) during sessions preceding success. What is common across infants is the large amount of fluctuation and the lack of a clear, single tendency: we might have expected, for instance, to observe an increase in the connection between rake and toy in the session preceding success, but this was not the case.

Thus, we found little evidence that gradual accumulation of knowledge about rake affordances leads to the ability to make the connection between rake and toy. There was frequent rake behavior but it did not gradually increase; nor was rake + toy behavior systematically preceded by frequent rake behavior.

***Learning from trial and error***. While the infants appeared not to have learned from their unexpected successes in C2 during the first session, we wondered if they learned from their errors in C4–C5. In other words, did they correct their movements after trying unsuccessfully to grasp the toy with the rake? There is some indication of this, since behavior S1, which reflects awkward or partly successful attempts to use the rake to obtain the toy (trial and errors), was more frequent in the first half of the first successful session (22.2%) than in the second half of the same session (17.5%), whereas S2 increased from 1.6 to 11.1% (see Video S3).

**Figure 8:**
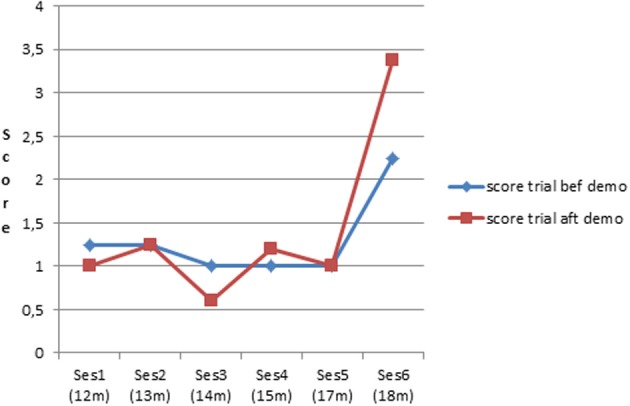
**Mean score on trial before and after demonstration in C4–C5**.

***Learning from demonstration by an adult***. Another mechanism to learn how to use a rake might be to observe others doing it. This would be a more economical method than trial and error. As mentioned above, in all sessions, after the first failure in C4 and C5, infants received a demonstration from either the parent or one of the experimenters (usually two demonstrations in a row). Infants clearly did not learn much from the adult's demonstration until late in the study. With only one exception (infant 1, session 3), none of the infants succeeded in retrieving the toy with the rake in C4 or C5 right after a demonstration before the sixth session. In addition, infant 1 did not repeat her success before the sixth session, either before or after demonstration. To check whether the behavior had been influenced by the demonstration despite not sufficing to lead to retrieval of the toy, we compared the level of performance, indexed by the obtained score, on the trials preceding and following demonstration for C4 and C5 considered together (see Figure 8). It can be seen that the score on trials just following demonstration did not differ greatly from the score of the trials preceding a demonstration until the last session. An ANOVA was performed on the score as a function of condition (×2, before and after the demonstration), and of session (×4, we choose to start at session 3 to be able to include infant 2) with repeated measures. It showed no main effect of condition, a significant and large main effect of session [*F*_(3, 12)_ = 31.3, *p* < 0.001; partial η^2^ = 0.89], and a significant and moderate condition x session interaction [*F*_(3, 12)_ = 7.5, *p* < 0.01; partial η^2^ = 0.65]. A *post-hoc* LSD test indicated that on the last session the score after demonstration differed significantly from the score before demonstration (*p* < 0.0001). Thus, infants started to benefit from demonstration relatively late, and not before 18 months.

In sum, when the toy was not inside the rake, infants started to use the rake to retrieve the toy between 16 and 20 months of age. Before that, they either explored the rake *per se* or focused on the toy, or to a lesser extent made some connection between rake and toy but apparently without the intention to retrieve the toy with the rake. When successes first appeared, they often were awkward and partial, or they happened after demonstration by an adult. But neither of these behaviors was observed during the first sessions. It could be that the capacity to correct inadequate motor planning (trial and error strategy), and the capacity to benefit from a demonstration (observational learning) require that the infant already have some intuition of the solution.

## Discussion

As noted in the introduction, few controlled experimental studies have considered in detail what mechanisms might underlie the acquisition of the use of a rake-like tool in infancy, and among these, only one is longitudinal. The essential conclusion from the existing studies is that spatial proximity, number of planning steps, and familiarity with the tool are factors that play a role. However these studies present no hypotheses about what drives progress in tool acquisition. In particular, they do not elucidate the question of whether the child learns to use the rake through progressive familiarization with use of the rake in interacting with other objects, or whether more or less sudden insight is involved.

Even though our conclusions are incomplete, the present longitudinal study makes some preliminary qualitative steps toward answering this question, and toward sketching out possible mechanisms underlying tool acquisition. The approach was to study a small number of infants, and analyse their detailed behavior at regular intervals. In this respect, the study differs from most contemporary approaches, and comes closer to the old, more qualitative observational methods of Piaget. As such the conclusions do not have the same statistical value as is usual in today's studies, but they provide valuable ideas for further work.

A first interesting point that our results brings to light concerns the interpretation of early successes observed in the literature in cases when there is no spatial gap between rake and toy.

### Early successes when there is no spatial gap

Our study confirms previous work showing that situations where there is no spatial gap between the rake and the toy, infants as young as 9–12 months can have little difficulty retrieving the toy (Bates et al., 1980).

But our results allow us to provide more insight into these early successes than has previously been coming forth.

First, with respect to the situation where the toy is attached to the tool: here our results clearly show that at 12 months (and most probably before that) children already know that they can move one part of a rigid object by moving another part: all infants succeeded as of the first trial and looked clearly at the toy from the start of their pulling movement. The result is compatible with extensive work using purely perceptual measures at even earlier stages of development (e.g., Spelke and Van de Walle, 1993).

But second, an important contrast exists with respect to the situation where the toy is contiguous but not attached: it can be touching or with a small spatial gap but within the trajectory of the rake. Here our results show quite distinctly that successes in such cases do not correspond to real understanding of the function of the rake as a tool, but to the fact that because of its spatial proximity, playing with the rake will likely cause the toy to move. Evidence that the infant had no notion that the rake would bring the toy closer is first: when the child moves the toy with the rake but not far enough to grasp it, the child will often not continue using the rake but stretch out with its hand to try to get the toy; and second, after a successful trial, in a subsequent trial an infant will often grasp the tool and move it around the toy before pointing to the toy (see Voulomanos, 2011, for other examples of u-shape developmental curves).

### Means-end behaviour

The conclusion from these considerations is thus that real understanding of the use of the rake as a tool only emerges in our data after about 18 months, and that early successes without a spatial gap do not correspond to proper understanding. This is consistent with our previous cross-sectional study (Rat-Fischer et al., 2013), but it raises the question of the relation to the literature on means-end behavior.

In the literature it is sometimes claimed (Willatts, 1999) that means-end behavior of various types is observed as early as 8 months, examples being given of the case of a cloth support, string, or of an extended tool like a stick, to obtain an out of reach object.

Our results lead us to ask whether success in such studies could be reinterpreted in a way similar to what we proposed for the rake task in the no-spatial-gap condition: Could it be that in many classical means-end tasks, successes before the second year of life were accidental, and due to the fact that any small motion of the cloth/string/tool will have tended to bring the object into motion, thereby drawing attention toward the object, causing the child to look at it, and then allowing the child to attain it by manual grasping. For instance, Willatts' (1999) experiment showing apparent clear presence of intentional cloth-pulling to retrieve a toy at 8 months can be re-interpreted as infants' having (1) an automatic cloth-pulling action which they put into play whenever confronted with the cloth/toy situation; and (2) having a larger attentional span in peripheral vision, and as a consequence being more likely to notice the toy moving and thus to look at it; and (3) having overall larger arm motions, thus making the cloth move further on every pull. These three mechanisms would result in coding as “intentional” (measured among others by probability of attaining the toy, probability of looking at it). A further suggestion that younger infants might not actually fully, practically, understand the function of the tool, can be got from experiments in which the infant is given the choice of several strings, or between different tools, in order to solve the task. In such situations it is known that children do not succeed immediately until well into the second year (Brown, 1990). Similarly, in one study on 14 infants aged 16 months, we also observed that infants rarely chose the correct string among a set of four when three were non-connected (Rat-Fischer et al., under revision). It can always be claimed that difficulty in such situations derives from confusion, attentional load, or goal/sub-goal competition induced by the visually more complicated set-up, but a more parsimonious account of the results when taken together with our present findings, might be that infants in fact do not have proper practical understanding of the notion of tool as a means to attain an object until about 18 months. Sommerville and Woodward's (2005) observation that 10-month old infants, as a group, do not “planfully” succeed in a cloth support task is consistent with these ideas. Note, however, that in the same study, the authors observed that when a rectangular box was substituted for the cloth, 10-month-old infants “planfully” succeeded in pulling the box to retrieve the toy, thereby demonstrating a sensivity to the causal structure.

The above re-interpretation of 8-month-old's successes at many classical means-end tasks, does not, we suggest, apply to our 12-month-old's success at C1 (toy attached to the rake). There we claim the children's behavior is truly intentional, reflecting knowledge that moving one part of the rake will move another part. This seems likely because the children were older, but also the data clearly demonstrate that all infants succeeded as of the first trial and looked clearly at the toy from the start of their pulling movement.

### Path to successful use of the tool when there is a spatial gap

A major purpose of the present study was to try to cast light on the process that leads to infants finally understanding the notion of tool. We were hoping that among the different behaviors, behavior T+R, that is, the behavior of bringing the rake into contact with the toy, would allow the infant to test the affordances of the rake and bring the child closer to understanding its functionality. We thus expected that T+R would generally increase before the child demonstrates success. Such findings would have been in line with Kahrs et al.'s (2012) observation of progress in the kinematics of banging movements between 7 and 14-months, that the authors considered as a pre-adaptation for later instrumental hammering, as well as with the observation of “non-random errors” and exploration of objects preceding tool-use in young animals (Meulman et al., 2014).

Curiously however this was not what we found. Taking together the data for all infants, we found that the T+R category of behaviors was not obviously correlated with subsequent successes at C4–C5, and occurred about equally often in all sessions preceding Sessions 4 and 5 where successes started occurring. Looking at the data individually for each infant also did not reveal any tendency for T+R or any other behavior to increase clearly just before success for any infant.

### Factors limiting success

One first limiting factor could have been that infants actually did not have the goal of retrieving the toy. However this does not seem to be the case. Even though some infants showed an interest in playing with the rake, the desire to get the toy was evident at some point in all sessions for all infants.

As already mentioned, in C4–C5 infants frequently pointed toward the toy as their first action. This pointing might be an example of pre-potent action patterns that young learners must inhibit in order to solve the problem (by using the tool). The difficulty of overcoming prepotent actions has been demonstrated with young animals (cf. review by Meulman et al., 2014). Inhibition of prepotent action patterns may be facilitated by maturation of the prefrontal cortex (Diamond and Gilbert, 1989).

Attentional limitations might be another limiting factor. In C4-C5 it is generally the case that after first trying to attain the toy by pointing toward it, infants lose interest and then switch their attention to the rake. However this attention shift does not imply that infants know that the rake can be used to get the toy. On the contrary, our evidence suggests that infants' goal is now purely to explore the rake for its own sake. After some tool exploration, infants then often revert to pointing toward the toy. It could be the case that dividing attention between the task at hand (how to retrieve the toy?) and the affordance of a novel object (what kind of actions can be done with a rake?) involves excessive cognitive load for the infant.

Another limiting factor to be considered is manual dexterity. It could be argued that physical inability to move the tool with an effective movement limits the chance of success. There are two reasons why we do not think this to be the case: first, once the infants started to try to use the rake to retrieve the toy, these partial successes were very rapidly followed by efficient successes, within the same session. In other words, after trying to use the rake to retrieve the toy, infants might be awkward for the first trial but corrected their error almost immediately. Second, in another study, pure visual exposure to a parent using the rake several times at weekly intervals, without the infant itself being allowed to manually manipulate the rake, was enough to significantly advance the age of success by between two weeks and two months (Somogyi et al., under revision).

### Lack of learning from observation in our study

Another piece in the puzzle that must be integrated into a theory explaining the emergence of rake use is our striking result on the effect of demonstration from an adult: infants were only able to profit from a demonstration precisely around the age when they would in any case be able to do the task spontaneously^1^. This is consistent with our previous cross-sectional study bearing on 60 infants, aged 14, 16, 18, 20, and 22 (Rat-Fischer et al., 2013) and with other work showing that proper understanding of the causal structure of means-end tasks in observational learning only matures in the second half of the second year (Meltzoff, 1995; Bellagamba and Tomasello, 1999; Huang et al., 2002).

However, it is in contrast to some other recent research which has investigated the ability of infants to solve means-end problems by observing an adult perform the task. In these experiments it was found that in certain means-end tasks, infants are able to profit from observation of a demonstration as early as 12 months (Provasi et al., 2001; Esseily et al., 2010; see Elsner, 2007, for a review). Such findings seem incompatible with our current finding that even after demonstration, infants were unable to succeed in using the rake until about 18 months.

As a way to explain the incompatibility, a possibility might be to claim that the particular materials employed by Esseily et al. and Provasi et al. had the property that even a fairly approximate imitation of the adult's demonstration would tend to lead to success. The means-end tasks used by these authors thus more closely resembled the *no spatial gap* conditions of our experiment, where infants were frequently successful because, however they moved the rake, the toy was likely to come closer. This is in contrast to the spatial gap conditions of our experiment, where a particular, precise form of raking motion is necessary for success.

To explain the ability to learn from demonstration from an adult in Esseily et al. and Provasi et al., we could then appeal to the fact that infants as early as 6 months have the capacity to *imitate* actions that they are shown (or have seen) (see Poulson et al., 1989; Elsner, 2007; Elsner et al., 2007, for reviews). Because of the relative simplicity of the tasks involved in Esseily et al. and Provasi et al., such imitation might then have led to higher success rates in the demonstration conditions. But under this hypothesis, these successes would not have corresponded to real understanding of the functionality of the means that led to success. In our experiment, where the task is somewhat more complex involving two stages (first grasping the rake, then adequately manipulating it), such imitation without understanding will not have led to success.

In conclusion, this longitudinal study of five infants learning how to use a rake reveals the interest (and difficulty!) of studying individual behaviors in a particular task over several months. No single type of behavior in our study seemed to lead systematically to success, leading us to suggest that many processes are involved. It may be that a variety of experiences involving familiarization with objects, exploration of object affordances, attentional factors, social cues, action planning, some of them associated with personal experience, others associated with brain maturation, each contribute small amounts of expertise that all come together fairly suddenly around 18 months to allow the child to understand that the rake can extend the body's range of action. There may be different routes to success and the mechanisms leading to success may differ from one infant to the next. An interesting question for future work will be to manipulate factors or conditions that allow infants to acquire tool use earlier than the second year—an example being our finding that simple visual exposure to the parent using the rake several times at weekly intervals alone can accelerate progress by many weeks (Somogyi et al., under revision). Future work should also include using different materials to check to what extent the children transfer their knowledge to different tools and different situations.

### Conflict of interest statement

The authors declare that the research was conducted in the absence of any commercial or financial relationships that could be construed as a potential conflict of interest.

## Footnotes

^1^Although we have very recently demonstrated that emphasizing the demonstrator's intentions may advance the age of success, cf. Esseily et al. (2013).

## Supplementary material

Video S1 **Infant 1, 15 months, C2: discarding the tool**.

Video S2 **Infant 3, 12 months, C4: hitting the toy with the rake**.

Video S3 **Infant 1, 18 months, C5: trial and error and observational learning before first full success**.
